# Hyperhomocysteinaemia in Behçet's Disease

**DOI:** 10.1155/2010/361387

**Published:** 2010-09-28

**Authors:** Amira Hamzaoui, Olfa Harzallah, Rim Klii, Silvia Mahjoub

**Affiliations:** Department of Internal Medicine, Fattouma Bourguiba University Hospital, 5000 Monastir, Tunisia

## Abstract

*Objectives*. The aim of this study was to investigate if hyperhomocysteinaemia is a contributive risk factor for the pathogenesis and the activity of Behçet's disease (BD). *Design and Methods*. Fifty four patients fullfiling the criteria of the International Study Group for BD were enrolled. Fifty healthy volunteers matched for age and sex with the BD group were included as a negative control group. Patients, with any condition that might affect plasma homocysteine concentration, were excluded. *Results*. Mean serum homocysteine concentration was significantly higher in patients with BD than in the healthy controls (*P* < .001), in patients with active disease (*P* = .04), and in masculine gender (*P* = .05). There was no significant difference between homocysteine level and clinical involvement. *Conclusions*. We demonstrated that plasma total homocysteine level (tHcy) is increased in BD and correlated with disease activity. No association was found between homocysteine levels and clinical involvement.

## 1. Introduction

Behçet's disease (BD) is a systemic inflammatory vasculitis with unknown etiology, which typically presents with recurrent orogenital ulceration, uveitis, and erythema nodosum. Other involvement may characterise the course of the disease: articular, vascular, central nervous system, and gastrointestinal involvements [1]. Thrombophlebitis, deep vein thrombosis, arterial obstruction, and aneurysms are the more frequent vascular damage in BD [2]. 

Vascular endothelial dysfunction represents the major pathogenesis abnormalities in BD. This was demonstrated by the reports of elevated serum concentrations of von Willebrand factor, plasminogen activator inhibitor-1, and thrombomodulin in patients with BD [3, 4].

Evidence from recent studies suggests that activated leukocytes may contribute to vascular injury in BD. Concentration of circulating pro-oxidants and lipid peroxidations products are elevated in BD [5].

Homocysteine (Hcy) is a sulfur-containing amino acid produced during the metabolism of the essential amino acid methionine in all cells through the normal methylation process. High levels are reported in the course of diabetes mellitus, hyperlipidaemia, end-stage renal failure, psoriasis, and inflammatory bowel disease [6–9]; it may also occur due to vitamin B 12 or folate deficiency [10] or secondary to treatment with some drugs, such as Methotrexate [11]. 

In the other hand, hyperhomocysteinaemia (HHCY) may cause lipid peroxidation, impaired vasomotor regulation, prothrombotic surface, and, therefore, vascular endothelial injury and atherothrombogenesis. 

The exact mechanisms linking homocysteine to endothelial disfunction are still unknown. Retrospective and prospective studies support an association between HHCY and increasing risk of cardiovascular disease, such as coronary arterial disease, cerebrovascular disease, and venous and arterial thrombosis [12].

During the last decade, and according to a limited number of studies, HHCY is now assumed to be an independent risk factor for venous thrombosis [13, 14] and for occular involvement in BD [15]. 

The major studies showed that vitamin supplementation with Vitamin B 12, Vitamin B 6, and/or acid folic was associated in a decrease in Hcy levels.

The aim of this study was to investigate whether HHCY is a risk factor for the development of activation in BD.

## 2. Material and Methods

### 2.1. Methods

Fifty four patients (34 men and 20 women; mean age 31.12 ± 8.9 years) with BD according to the criteria of the International Study Group for Behçet's disease and 50 matched healthy volunteers (21 men and 29 women; mean age 37.38 ± 10.3 years) were enrolled in this prospective study. The detailed clinical characteristics were recorded for each patient.

 The exclusion criteria were diabetes mellitus, psoriasis, chronic hepatitis, chronic alcoholism, renal failure, and pregnancy. Patients were considered active according to the clinician's impression and in the case of the existence of two or more symptoms with worsening of clinical symptoms and lack of wellbeing at the time of the study.

### 2.2. Measurement of Homocysteine

The blood samples were collected in ethylenediaminetetraacetate (EDTA)—containing tubes after 12 h of fasting; they were kept in on ice in order to prevent homocysteine formation and leakage from erythrocytes, and were centrifuged immediately at 2000 × g for 10 minute at room temperature. Hcy was measured by high-performance liquid chromatography. HHCY was defined as a serum t-Hcy level > 15 *μ*mol/L.

### 2.3. Statistical Analysis

SPSS for Windows version 10.0 was used to analyse the data. In the statistical evaluation of the data, the independent samples *t*-test (Student's *t*-test) was used to compare the mean's of the two groups. Correlation analysis was performed with the Pearson correlation test *P* < .05 was accepted as significant (confidence interval = 95%).

## 3. Results

The cumulative clinical characteristics of BD patients are shown in Table 1. Demographic data of all groups are summarised in Table 2. The mean serum Hcy concentrations in each group are shown in Table 3. 

HHCY was significantly higher in BD patients than in controls (55.5% versus 8%; *P* < .01) and in active patients than in inactive (56% versus 43%; *P* = .05). No significant correlation was found between serum Hcy concentration and clinical features such as thrombotic events (*P* = .5), uveitis (0.08), retinal vasculitis (*P* = .06), and neurological involvement (*P* = .4). HHCY was significantly higher in patients with arthritis: 87.5% versus 52.17%; *P* = .05.

## 4. Discussion

BD is a vasculitic systemic syndrome with endothelial dysfunction [3, 4]. The mechanism of vascular involvement has not yet been clearly delineated. HHCY can be mild, moderate, or severe elevation of tHcy in blood or serum. Recent Data expanded the spectrum of the possible pathogenic implication of HHCY in the course of auto-immune and vasculitis disease: systemic and retinal vascular occlusive, thrombogenesis, and so forth.

Several effects induced by HHCY on vascular cells have been described: diminished nitric oxyde release from endothelial cells, producing of superoxyde and hydrogen peroxide, increased reactive oxygen species and thromboxane A2 formation in human platelets, potentiation of low density lipoprotein oxidation, and stimulation of smooth muscle cells proliferation.

These features changes coagulation factor so as to encourage blood clot formation with aggregated platelets and modified the adhesive properties of endothelial cells [24].

Elevated tHcy level has been shown to be a risk of myocardial infarction [25], stroke [26], and diabetes melitus. It may also increase the risk of retinal vascular diseases, such as retinal artery, retinal vein thrombosis, and occlusion [27, 28]. 

Our study showed that serum tHcy levels were significantly higher in whole BD patients than in the healthy controls, and in active patients. However, no significant difference was found between tHcy levels and clinical features. Some studies reported that HHCY is associated with BD. These studies are summarized in Table 4.

Others studies reported normal homocysteine level in BD patients [14, 29]. Hyperhomocysteinaemia was significantly higher in our male patients. It was found in the studies of Ates et al. [18] and Feki et al. [23]. These studies are summarized in Table 5. Our present results showed that serum tHcy levels and HHCY were higher in the active patients than those of the inactive patients and the control subjects. It was demonstrated by Feki et al. [23], Ates et al. [18], Sarican et al. [21], and Aksu et al. [13].

When correlated with occular involvement, the tHcy level were higher but not significantly in our BD patients with retinal vasculitis (21.09 ± 7.8 versus 16.9 ± 6.9 *μ*mol/L; *P* = .06) and uveitis (20.25 ± 7.8 versus 16.69 ± 6.7 *μ*mol/L; *P* = .08). 

Er et al. [15] found a significant correlation between tHcy level and ocular BD (18.25 ± 4.2 versus 13.53 ± 3.34 *μ*mol/L; *P* = .001); in addition, ocular BD had significantly higher serum endotheline-1 concentrations when compared with nonocular disease (19.17 ± 5.02 versus 15.62 ± 2.48 *μ*mol/L; *P* = .003). 

Okka et al. [30] showed that tHcy level was significantly higher in BD patients with uveitis when compared to inactive patients (*P* = .029). This correlation was found in others studies [19, 20, 23].

Furthermore, serum tHcy levels among ocular patients with observed retinal vasoocclusion were significantly higher than ocular patients without such occlusion, suggesting that thrombogenetic tendency is the main features for Hcy [31]. Aflaki and all found no significant correlation between serum Hcy level in patients with and without eye involvement (18.19 ± 9.21 versus 16.59 ± 9.51 *μ*mol/L; *P* = .31) [32].

Relation between retinal vascular occlusive disease and elevated levels of tHcy was assessed in several studies. Cahill et al. concluded that elevated tHcy is an independent risk factor for retinal vascular occlusive disease. In their study of 87 cases with retinal vascular disease (26 cases of retinal artery occlusion, 40 cases with central retinal vein occlusion, and 21 cases of branch retinal vein occlusion) compared with 87 age matched controls, mean tHcy levels were significantly higher in all disease groups [33]. 

HHCY is a well-known risk factor for the development of thrombosis. The role of HHCY as a contributing risk factor for thrombosis in BD was investigated in some studies. 

Aksu et al. [13] reported that patients with BD and a histories of thrombosis had significantly higher Hcy levels than those without thrombosis (15.3 ± 6.2 versus 9.3 ± 3.1; *P* = .0001). Lee et al. [14] suggested that patients with histories of thrombosis had also significantly higher levels of tHcy. When studying 45 patients with BD, Ates et al. [18] found that the 29 BD patients with vascular involvement had a significant higher tHcy levels when compared to controls (*P* = .001) and nonvascular BD (*P* < .01). The same results were found by Yesilova et al. [19]. 

No association was found between Hcy levels and vascular involvement in our BD patients. The same results were reported by Feki et al. [23] and Korkmaz et al. [16]. In a recent paper, Ricart et al. [34] found no difference of Hcy levels in patients with and without thrombosis. 

In the recent study conducted by Durmazlar et al., serum levels of Hcy in active BD patients with vascular involvement were found significantly increased when compared in patients with active BD without vascular involvement (19 ± 39.25 versus 13.7 ± 26 *μ*mol/L; *P* < .05). In addition, serum levels of Hcy in inactive BD with vascular involvement were found significantly increased compared to inactive BD (11.94 ± 18 versus 6.43 ± 16 *μ*mol/L; *P* < .005) [35].

Recently, Gönül et al. found no differences in tHcy levels between BD patients, patients with recurrent aphthous stomatitis, and controls [36].

In our study, we found a significant correlation with tHcy levels and arthritis (*P* = .05). Yesilova suggested that tHcy levels may be related to markers of inflammation; the depletion of folate could result from overdemand [19].

Durmazlar et al. found a significant positive correlation between serum Hcy and Tumor Necrosis Factor-*α* (TNF-*α*) levels (*r* = 0.89, *P* = .00), CRP (*r* = 0.645, *P* = .00), and ESR (*r* = 0.561, *P* = .00); Hcy appears to be the best predictor of TNF-*α* and positively correlated with inflammatory markers.

The mechanism of Hcy associated activation of BD and endothelial toxicity is not fully understood. It may be in relation with increased oxidative stress, platelet activation and atherogenesis by oxidative injury, increased adhesiveness, enhanced coagulability, and vascular matrix damage. 

Another mechanism is the participation of the oxidant/antioxidant balance; the decreased activity of endothelial derived NO leads to vasoconstriction, platelet aggregation, and monocyte adhesion. A changed in favour of oxidants occurs, and this imbalance may have a role in the pathogenesis and/or activity of BD [37]. 

It has been shown that concentrations of circulating pro-oxidants and lipid peroxidation products were elevated in patients with BD [5, 38], although the relationship between oxidative stress mechanisms and vascular injury in patients with BD has not been elucidated. It is known that Hcy generates superoxide and hydrogen peroxide, both of which have been linked to endothelial damage. Therefore, enhanced endothelial toxicity caused by elevated tHcy may play an important role in the pathogenesis in BD, and may cause the activation of the disease.

A second hypothesis is that Hcy promotes the clotting cascade via several action; it inhibits the expression and activation of thrombomodulin, which is a cofactor for protein C activation, activation of coagulation factor V, and increasing smooth muscle cells proliferation. It also suppresses the anticoagulant effect of antithrombin III [39]. An association with the factor V of Leiden has also been reported.

In conclusion, we demonstrated that serum tHcy levels are increased in BD and correlated with disease activity. Because HHCY is a treatable risk factor, measurement and monitoring of tHcy levels may be valuable index in the evaluation of patients with BD especially in active stage.

## Figures and Tables

**Table 1:** Cumulative clinical characteristics of patients with Behçet's disease.

	*N*	Percent
Oral ulceration	54	100
Genital ulceration	49	90
Papulopustular eruption	46	85.2
Pathergy positivity	18	41.9
Arthritis	8	14.8
Uveitis	20	37
Retinal vasculitis	15	27.7
Vascular involvement	21	38.8
Deep vein thrombosis	15	27.7
Arterial aneurysm	4	7
Pulmonary embolism	7	13
Central nervous system involvement	20	37

**Table 2:** Demographic data (mean ± SD).

	BD (*n* = 54)	Healthy controls (*n* = 50)
Age (years)	31.12 ± 8.9	37.38 ± 10.3
Male/Female	34/20	21/29

**Table 3:** Serum homocysteine concentration.

Groups	Homocysteine concentration (*μ*mol/L)
	Mean ± S.D (min − max)
BD group (*n* = 54)	18.07 ± 8.9^a^
Healthy group (*n* = 50)	11.2 ± 6.5
Active group (*n* = 17)	20.7 ± 8^b^
Inactive Group (*n* = 37)	16.6 ± 6.5

^a^Higher than in the healthy group; *P* < .01.

^b^Higher than in inactive group; *P* = .05.

**Table 4:** Homocysteine and Behçet's disease.

Author (year)	Patients/Controls	Homocysteine concentration in BD patients (*μ*mol/L)	Homocysteine concentration in healthy (*μ*mol/L)	*P*
Aksu et al. (2001) [13]	84/36	11.5 ± 5.3	8.8 ± 3.1	<.01
Er et al. (2002) [15]	43/25	15.83 ± 4.44	7.96 ± 6.3	<.001
Korkmaz et al. (2002) [16]	74/35	16.08 ± 7.5	12.9 ± 6.3	<.03
Houman et al. (2003) [17]	59/59	13.3 ± 6.8	10.9 ± 2.4	<.01
Ates et al. (2003) [18]	45/40	11.3 ± 4.3	9.1 ± 2.5	<.01
Yesilova et al. (2004) [19]	32/20	14.86 ± 4.26	10.78 ± 2.28	<.001
Ozdemir et al. (2004) [20]	31/30	14.5 ± 2.6	9.2 ± 2.8	<.0001
Our study (2005)	54/50	18.07 ± 8.9	11.2 ± 6.5	<.01
Sarican et al. (2007) [21]	64/26	11.7 ± 4.6	8.7 ± 2.8	<.01
Ozcan et al. (2007) [22]	23*/21	12.0 ± 3.3	10.7 ± 2	<.03

*Active disease.

**Table 5:** Homocysteine and gender.

Authors (years)	Hcy (*μ*mol/L) Male	Hcy (*μ*mol/L) Female	*P*
Ates et al. (2003) [18]	12.8 ± 4.5	8.3 ± 1.6	<.001
Feki et al. (2004) [23]	11.5 (7.7–20.6)	9.7 (6.0–15.3)	—
Our study (2007)	19.37 ± 7.6	15.69 ± 6.2	.05
